# Colonic Diverticulosis and Uncomplicated Diverticulitis Are Associated With a Lower Not Higher Risk of Mortality When Confounding Factors Are Held Constant

**DOI:** 10.1111/jgh.16928

**Published:** 2025-03-17

**Authors:** Raquel A. Cameron, Michael P. Jones, Guy D. Eslick, Nicholas J. Talley

**Affiliations:** ^1^ College of Health, Medicine and Wellbeing University of Newcastle Newcastle Australia; ^2^ NHMRC Centre for Research Excellence in Digestive Health Newcastle Australia; ^3^ Hunter Medical Research Institute Newcastle Australia; ^4^ School of Psychological Sciences Macquarie University North Ryde Australia

**Keywords:** mortality, diverticulosis, diverticulitis, diverticular disease, colon, gastroenterology

## Abstract

**Background and Aim:**

The association between colonic diverticulosis, diverticulitis, and mortality is controversial. This study evaluated the association between diverticular disease and mortality over a prolonged period in a GP cohort.

**Methods:**

GP records were sourced from the United Kingdom medical database (THIN). Diverticulosis and diverticulitis were identified via Read codes. The overall patient cohort (*n* = 1 274 260) included patients with colonic diverticula (*n* = 39 521 [3.1%], mean age 54) and no diverticula (control group) (*n* = 1 234 739 [96.9%], mean age 38). Poisson regression estimated relative rates, and durational time at risk and survival probability were calculated.

**Results:**

Colonic diverticula are associated with an increased mortality risk when compared with nondiverticular patients (OR = 1.89, 95% CI 1.84–1.94; *p* < 0.001). However, controlling for age, sex, and potential confounding variables yielded a decreased mortality risk overall for colonic diverticula patients (HR = 0.66, 95% 0.64–0.6; *p* < 0.001). When the diverticulitis cohort is separated into uncomplicated and complicated, increased mortality is observed in both uncomplicated diverticulitis (HR = 0.64, 95% CI 0.61–0.66; *p* < 0.001) and complicated diverticulitis (HR = 1.14, 95% CI 1.02–1.28; *p* = 0.024), but on controlling for confounding, there is a decreased risk of mortality for uncomplicated diverticulitis (HR = 0.65, 95% CI 0.63–0.66; *p* < 0.001) but almost two times increased mortality risk for complicated diverticulitis (HR = 1.18, 95% CI 1.05–1.32; *p* = 0.006).

**Conclusions:**

In this large UK GP sample, controlling for age, sex, and comorbidities, patients with uncomplicated diverticula are associated with a lower mortality risk. However, complicated diverticulitis still carries two times the risk of mortality than those with no or uncomplicated colonic diverticula.

## Graphical abstract

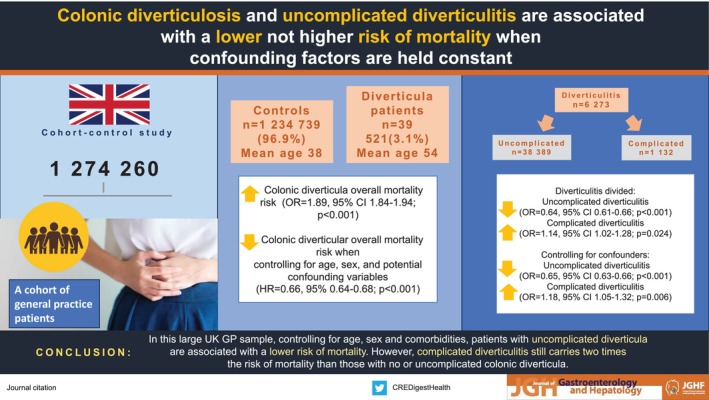

**Abbreviations**
CI/s: confidence interval/s
HR: hazard ratio
GP/s: general practice/practitioners
NHS: National Health Service
OR/s: odds ratio/s
SD: standard deviation
THIN: The Health Improvement Network
WHO: World Health Organization

## 1 Introduction

Colonic diverticulosis is a common disease [1] identified across Westernized countries [2], yet it is primarily an asymptomatic pathologic condition [3, 4]. An advancing age‐associated disease [1], colonic diverticulosis affects an estimated 30% of individuals aged 60 and over, increasing to 70% or higher over the age of 80 years [3, 4]. Of these patients with known asymptomatic diverticula, it has been reported that anywhere between 4% and 25% become symptomatic [2, 3, 4]. It is estimated that 10%–15% [5] of symptomatic patients eventually present to an emergency department with diverticulitis or its complications, notably perforation, abscesses, fistula, stricture, or bleeding [2, 5, 6].

Recent reports suggest that not only is the incidence of colonic diverticulitis increasing [2, 7] but so too are hospitalization admissions for complicated diverticulitis, as are emergency surgical interventions [6, 8, 9]. There is an increasing worldwide disease burden of diverticulitis in terms of morbidity and mortality, which impacts the healthcare economy [2, 6]. In the United Kingdom, admission rates for acute colonic diverticulitis rose between 1996 and 2006 from 0.56 to 1.20/1000/year, with a reported 2.28‐fold increase in perforation admissions [2], equating to approximately 12 000 emergency bowel resections per year. In the United Kingdom, perforated diverticular disease alone has an important associated short‐term (8.4%) and long‐term mortality rate (14.5%) [2]. The exact cost burden to the UK National Health Service (NHS) is unreported [2]. A recent Australian study [7] concluded that complicated diverticulitis is currently increasing the length of hospital stay for acute diverticulitis patients (80%) despite conservative care management [7]. Less is known about how colonic diverticular disease impacts mortality in general primary care practices [10].

Studies of mortality in patients with colonic diverticular disease tend to be short‐term, focusing on diverticulitis complications [4, 8], and studies of long‐term mortality are rare and often undertaken without controls of comparable disease‐free individuals [4]. Therefore, it is controversial if colonic diverticular disease is associated with increased mortality. A recent Swedish cohort study [3] suggested that overall mortality in patients with colonic diverticular disease may be increased in the general population (hazard ratio (HR) 1.34, 95% confidence interval (CI) 1.32–1.36).

This study sought to evaluate whether colonic diverticular disease is associated with excess mortality over a prolonged period in a UK nationwide general practice cohort. As patients are required to register with a general practice in the United Kingdom, the cohort includes healthcare seekers and those not otherwise seeking healthcare. Potentially confounding variables of age at first contact with the medical practice and sex at birth, as well as comorbid diseases, were further controlled for in estimating the association between diverticular disease and mortality. Any association with excess mortality was also explored by evaluating uncomplicated and complicated diverticulitis mortality.

## 2 Methods

### 2.1 Data Acquisition

Study subjects were drawn from UK general practices with longitudinal patient data captured from electronic medical records via The Health Improvement Network (THIN) [11, 12]. This UK primary care research database contains anonymized electronic health records from over 850 general practices (GPs) (via the VISION clinical system; cegedim, UK), with around 10 million active patients [13]. THIN collects raw data from practices, queries possible data errors, and standardizes fields related to diagnoses, prescriptions, and referrals for comparability across practices [14]. THIN follows an equivalent methodology to the General Practice Research Database [15], which is representative of the UK general population with respect to demographics, significant condition prevalence and mortality rates within the United Kingdom [14]. The validity of THIN gastrointestinal disorder diagnoses (peptic ulcer disease, colorectal cancer, gastrointestinal bleeding) has been undertaken and was robust [12]. While diverticular disease was not included in the validation work, which may be a limitation, other endoscopic diagnoses were validated. This procedure has been approved by the UK National Health Services' South‐East Multicentre Research Ethics Committee (Ref: 20/SC/0011; March 20, 2020). Funding to obtain access to the data was provided by a grant from AusEE Inc (Australia).

### 2.2 Patient Initial Identification and Diagnosis Codes

The original patient cohort (*n* = 1 274 260) was selected from the THIN dataset based on having one or more of the following diagnoses: irritable bowel syndrome (IBS) (*n* = 139 194; 10.92%), functional dyspepsia (FD) (*n* = 232 107; 18.22%), or chronic constipation (*n* = 12 297; 0.97%), in their medical record. A cohort of controls with no gastrointestinal disease record was also identified (*n* = 36 435; 2.86%). Inclusion criteria also required a follow‐up period of at least 10 years of GP medical records. This dataset of digestive gastrointestinal–brain interaction (DGBI) healthcare seekers was chosen to represent an overall gastrointestinal healthcare seeking sample, with the rationale that this cohort would be a reasonable cohort to answer digestive health hypotheses. DGBIs are not associated with increased mortality outcomes but often overlap with colonic diverticulosis and diverticular disease diagnosis [16, 17, 18]. Patient covariate and comorbidity data were extracted by searching the dataset using the variable parameters employed for this study (Supporting Information S1).

## 3 Colonic Diverticulosis and Diverticular Disease Definition

From this retrospective cohort, colonic diverticulosis and diverticulitis (also referred to as “any diverticula”) were defined by Read codes that corresponded to Medcodes (K57 and K57.90 [diverticular disease and diverticulosis]; K57.30, K57.31, and K57.91 [diverticulosis of the colon, large intestine, including unspecified and nos, bleeding diverticulosis]; K57.20 and K57.80 [perforated diverticulum of the colon, large intestine, including unspecified and nos, diverticular abscess]); these codes are used across the United Kingdom for record reproducibility, allowing follow‐up beyond the primary care setting [14]. From the 1.27 million patient records obtained from THIN, patients who did not have one of the Read or Medcodes corresponding to diverticula were included within the control cohort. Further analysis separated uncomplicated and complicated diverticulitis. Complicated diverticulitis was defined as cases of diverticula bleeding (*n* = 56; died 37.5%), perforation (*n* = 458; died 23.8%), abscess (*n* = 249; died 22.9%), or fistula (*n* = 385; died 29.1%).

### 3.1 Determination of First Contact and Length of Follow‐Up

First contact was recorded as the first date the patient registered with the practice. Follow‐up was reported as the time from first contact until a reported death date, or the patient was no longer registered with that GP. Patients' age at first and last contact (or death) was also recorded. Cause of death information was not provided in the available data set.

### 3.2 Statistical Analysis

This study aimed to compare the mortality rate of patients with colonic diverticulosis, diverticulitis, or neither within a follow‐up period. Further, we aimed to determine the mortality percentages of the cohort who were reported as alive or deceased after the follow‐up period.

The analysis initially employed logistic regression to determine the risk of mortality in the cohort with a diagnosis of either diverticulosis or diverticulitis if time‐at‐risk and potential confounding variables are ignored. This analysis is reported in terms of odds ratios (OR) and 95% confidence interval (CI), as well as two‐tailed *p* values (*p*). This might mimic what a physician might observe in their clinical practice. To account for time‐at‐risk of mortality, we also utilized Cox's proportional hazards regression by considering time until either death or the end of the medical record. Patients remaining alive at the end of their medical records were treated as right censored. This analysis is reported in terms of HR, 95% CI and *p* value.

We further sought to determine if any associated excess mortality risk is attributed to confounding of age at first contact, sex at birth, and comorbidity variables (Crohn's disease, ulcerative colitis, esophagitis, *Helicobacter pylori* infection, diabetes types 1 and 2, appendicitis, chronic obstructive pulmonary disease [COPD], obesity, dyslipidemia, alcohol‐related disease, eosinophilic pulmonary disease [EPD], dementia, lung cancer, stroke, chronic kidney disease [CKD], liver disease, gastric cancer, colorectal cancer, and other cancers), by controlling for these variables in the Cox regression model.

The analyses to determine the risk of mortality and excess mortality risk were further reported on the diverticulitis cohort by splitting the patient group into uncomplicated and complicated diverticulitis. Kaplan–Meier estimation was undertaken by calculating the survival probability of uncomplicated and complicated subgroups against the control group (no diverticulosis).

## 4 Results

### 4.1 Patient Cohort

Cohort baseline demographics are reported in Table 1. Overall, the THIN database supplied 1 274 260 patients meeting the inclusion criteria. The cohort was composed of a patient cohort (all diverticula) (*n* = 39 521 [3.1%], mean age 54) or no diverticula (*n* = 1 234 739 [96.9%], mean age 38) (control comparison group). Patients with colonic diverticula were further subgrouped into patients with uncomplicated diverticula (combined colonic diverticulosis and uncomplicated diverticulitis) (*n* = 38 389 [3.0%], mean age 55 years) and complicated diverticulitis (*n* = 1132 [0.1%], mean age 53). The overall cohort at baseline was predominantly female (*n* = 722 913; 56.7%). Patients' first contact with their GP occurred predominantly before reaching 30 years of age (*n* = 378 919; 31.3%), with patients aged 80 years less likely to have their first GP appointment for diverticula disease at that age (*n* = 18 002; 1.5%), with an overall duration of follow‐up averaging 16 years (SD 7.0) (Table 1). Cumulatively, patients aged below 30–59 years accounted for 82.6% of the study population at first GP contact. Follow‐up person‐years were calculated for patients with uncomplicated diverticulitis (17 356 021 person‐years), complicated diverticulitis (709 067 person‐years), or neither (20 222 person‐years).

**TABLE 1:** Baseline demographics of the entire cohort (*n* = 1 274 260). Sex ratios by number (*n*) and percentage (%), age ranges for first contact recorded in general practice (GP) records, subgrouped further by the mean and standard deviation (SD) of those with diverticulosis or diverticulitis, and those without. The duration of GP records held in terms of patient numbers (*n*) and %, along with the mean (with SD) in years of follow‐up duration.

Sex, *n* (%)	
Female	722 913 (56.7)
Male	551 347 (43.3)
Age range at first contact, *n* (%)	
< 30	378 919 (31.3)
30–39	235 019 (19.4)
40–49	207 858 (17.2)
50–59	177 634 (14.7)
60–69	127 611 (10.5)
70–79	65 535 (5.4)
80+	18 002 (1.5)
Patients identified, mean age AFC (SD)	
With uncomplicated diverticula	55 (3.0)
With complicated diverticulitis	53 (0.1)
Without diverticulosis/itis (controls)	38 (96.9)
Duration of GP record, *n* (%)	
1–5 years	91 854 (7.6)
6–10 years	124 010 (10.2)
11–15 years	348 545 (28.8)
15+ years	646 169 (53.4)
Duration of follow‐up in years, mean (SD)	16 (7.0)

### 4.2 Demographics and Comorbidities in Diverticular Disease

Tables 2 and 3 tabulate mortality and comorbidities by age and sex (female vs. male). Table 2 describes the whole cohort split into those with and those without any recorded history of diverticula. Table 3 describes the whole cohort split into those with any recorded colonic diverticula history, identified as uncomplicated or complicated diverticulitis.

**TABLE 2:** Demographics and comorbidities of the entire cohort according to any diverticula (any recorded history of colonic diverticula). Logistic regression of patient demographics: sex (female), those who died during the period of follow‐up, and a list of potentially related comorbidities were tabulated by number (*n*) of patients and the percentage (%) of the cohort, with statistical significance (*p* value) for the division of the whole cohort (*n* = 1 274 260) into subgroups of those without diverticular disease indicated, and those with records indicating a diagnosis of diverticular disease.

	No diverticula (%)	Any diverticula (%)	*p*
*n*	1 234 739 (96.9)	39 521 (3.1)	
Age at 1^st^ contact, mean (SD)	38.33 (SD 20.7)	53.7 (SD 14.04)	< 0.001
Sex (F vs. M)	699 846 (56.7)	23 067 (57.4)	< 0.001
Died	112 451 (9.1)	6285 (20.6)	< 0.001
Obesity	2180 (0.2)	132 (0.3)	< 0.001
Alcohol‐related	9633 (0.8)	468 (1.4)	< 0.001
Dementia	8053 (0.7)	656 (3.2)	< 0.001
Eosophagitis	97 060 (7.9)	8307 (17.7)	< 0.001
Lung cancer	1549 (0.1)	463 (1.6)	< 0.001
COPD	4718 (0.4)	535 (1.5)	< 0.001
EPD	56 (< 0.1)	5 (< 0.1)	0.059
Dyslipidemia	40 485 (3.3)	3442 (7.8)	< 0.001
Stroke	18 489 (1.5)	1607 (8.8)	< 0.001
Diabetes type I	5871 (0.5)	167 (0.8)	0.302
Diabetes type II	83 749 (6.8)	6721 (16.5)	< 0.001
CKD	4718 (0.4)	535 (3.0)	< 0.001
Appendicitis	19 323 (1.6)	747 (3)	< 0.001
Liver disease	2921 (0.2)	256 (0.13)	< 0.001
*Helicobacter pylori*	10 139 (0.8)	838 (3.3)	< 0.001
Gastric cancer	27 122 (2.2)	2336 (13.2)	< 0.001
Crohn's disease	3193 (0.3)	239 (1.6)	< 0.001
Ulcerative colitis	3494 (0.3)	337 (0.8)	< 0.001
Colorectal cancer	1549 (0.1)	463 (0.8)	< 0.001
Other cancer	40 720 (3.3)	3342 (7.8)	< 0.001

Abbreviations: CKD = chronic kidney disease; COPD = chronic obstructive pulmonary disease; EPD = eosinophilic pulmonary disease; F vs. M = female versus male; *n* = number; SD = standard deviation.

**TABLE 3:** Demographics and comorbidities of the entire cohort according to any recorded history of uncomplicated diverticular disease or complicated diverticular disease at baseline. Logistic regression of patient demographics: sex (female), those who died during the period of follow‐up, and a list of potentially related comorbidities were tabulated by number (*n*) of patients and the percentage (%) of the cohort, with statistical significance (*p* value) for the division of the whole cohort (*n* = 1 274 260) into subgroups of those without diverticular disease indicated, and those with records indicating a diagnosis of diverticular disease.

	No diverticula (%)	Uncomplicated diverticular disease (%)	Complicated diverticular disease (%)	*p*
*n*	1 234 739 (96.9)	38 389 (3.0)	1132 (0.1)	
Age at 1^st^ contact, mean (SD)	38.33 (SD 20.7)	54.79 (SD 12.80)	52.61 (SD 15.28)	< 0.001
Sex (F vs. M)	699 846 (56.7)	22 428 (58.4)	639 (56.4)	< 0.001
Died	112 451 (9.1)	5995 (15.6)	290 (25.6)	< 0.001
Obesity	2180 (0.2)	127 (0.3)	5 (0.4)	< 0.001
Alcohol‐related	9633 (0.8)	470 (1.2)	18 (1.6)	< 0.001
Dementia	8053 (0.7)	639 (1.7)	17 (1.5)	< 0.001
Eosophagitis	97 060 (7.9)	8147 (21.2)	160 (14.1)	< 0.001
Lung cancer	1549 (0.1)	458 (1.2)	5 (0.4)	< 0.001
COPD	4718 (0.4)	516 (1.3)	19 (1.7)	< 0.001
EPD	56 (< 0.1)	5 (< 0.1)	0 (< 0.1)	0.06
Dyslipidemia	40 485 (3.3)	3365 (8.8)	77 (6.8)	< 0.001
Stroke	18 489 (1.5)	1553 (4.0)	54 (4.8)	< 0.001
Diabetes type I	5871 (0.5)	163 (0.4)	4 (0.4)	0.302
Diabetes type II	83 749 (6.8)	6541 (17.0)	180 (15.9)	< 0.001
CKD	4718 (0.4)	516 (1.3)	19 (1.7)	< 0.001
Appendicitis	19 323 (1.6)	699 (1.8)	48 (4.2)	< 0.001
Liver disease	2921 (0.2)	248 (0.6)	8 (0.7)	< 0.001
*Helicobacter pylori*	10 139 (0.8)	826 (2.2)	12 (1.1)	< 0.001
Gastric cancer	27 122 (2.2)	2253 (5.9)	83 (7.3)	< 0.001
Crohn's disease	3193 (0.3)	209 (0.5)	30 (2.7)	< 0.001
Ulcerative colitis	3494 (0.3)	329 (0.9)	8 (0.7)	< 0.001
Colorectal cancer	1549 (0.1)	100 (0.3)	3 (0.3)	< 0.001
Other cancer	40 720 (3.3)	3260 (8.5)	82 (7.2)	< 0.001

Abbreviations: CKD = chronic kidney disease; COPD = chronic obstructive pulmonary disease; EPD = eosinophilic pulmonary disease; F vs. M = female versus male; *n* = number; SD = standard deviation.

### 4.3 Diverticular Association With Mortality

**FIGURE 1:**
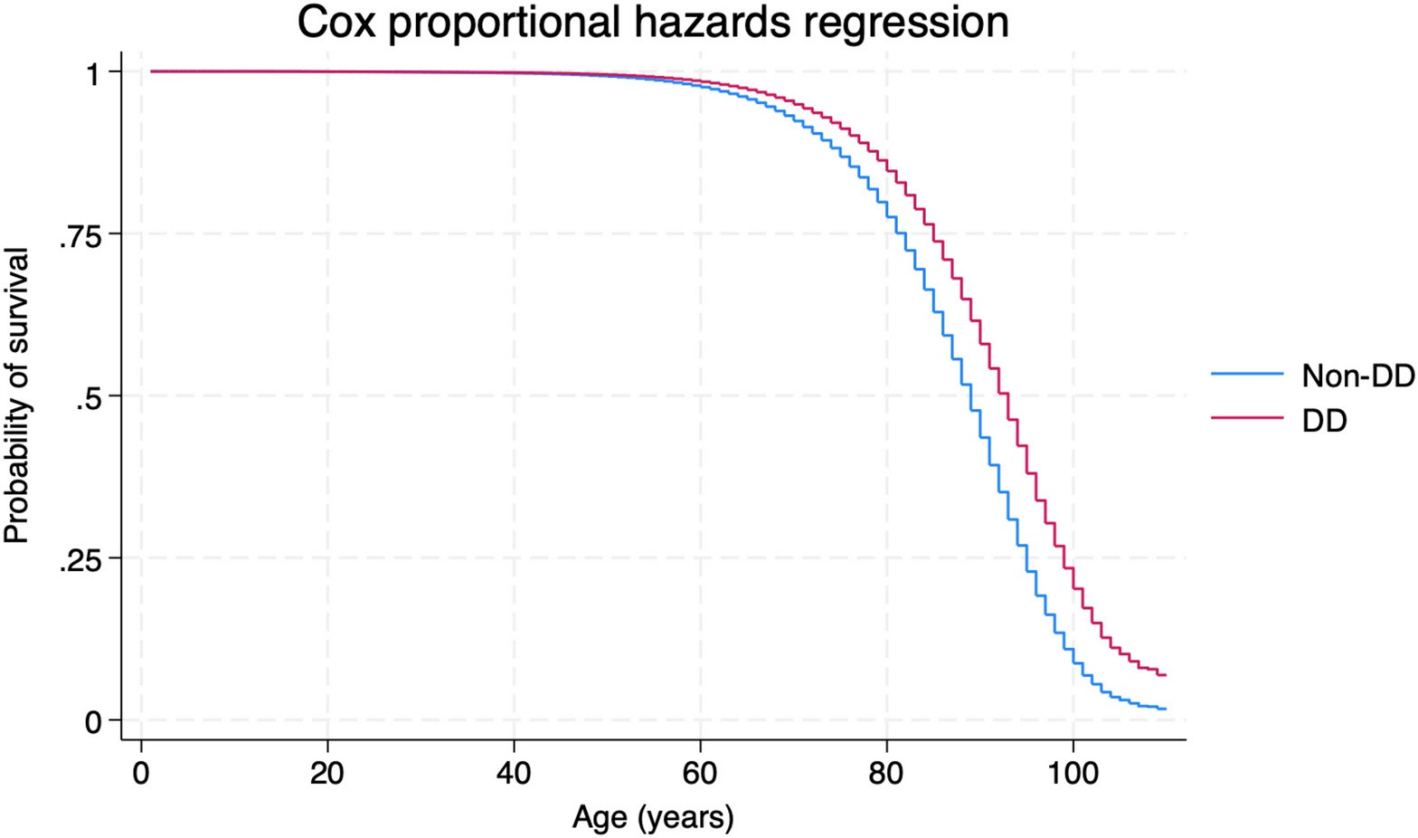
Time‐at‐risk for patients with diverticular disease. Time‐at‐risk is considered in a Cox proportional hazards regression model. The hazard ratio identified a reduced risk of mortality in patients with diverticular disease (red) against those without (non‐DD, blue). This is graphed according to age in years against the probability of survival (0–1).

Ignoring potential confounding variables and time at risk, any medical record of diverticular disease is associated with an increased mortality risk (15.9%). When compared with those with no diverticula (9.1.%), the odds ratio for mortality predicted colonic diverticula is OR = 1.89 (95% CI 1.84–1.94; *p* < 0.001). However, when time‐at‐risk is considered in a Cox regression model, the hazard ratio identified a reduced risk of mortality with diverticular disease (HR = 0.66, 95% 0.64–0.67; *p* < 0.001), which is clearly illustrated in the survival plot in Figure 1. Controlling for age at first contact and sex yields a similar hazard ratio estimate (HR = 0.63, 95% 0.61–0.64; *p* < 0.001). Further univariate analysis for all other potentially confounding variables in Table 2 still yields a similar hazard ratio estimate (HR = 0.66, 95% 0.64–0.68; *p* < 0.001).

Additionally, when the diverticulitis cohort is separated into uncomplicated and complicated diverticulitis groups, ignoring potential confounding variables, the mortality risk for simple diverticulitis was calculated as 15.6% and complicated diverticulitis as 25.6% (both *p* < 0.001). The mortality for patients with simple diverticulitis (HR = 0.64, 95% CI 0.61–0.66; *p* < 0.001) and complicated diverticulitis (HR = 1.14, 95% CI 1.02–1.28; *p* = 0.024) was increased when compared with the nondiverticular controls.

**FIGURE 2:**
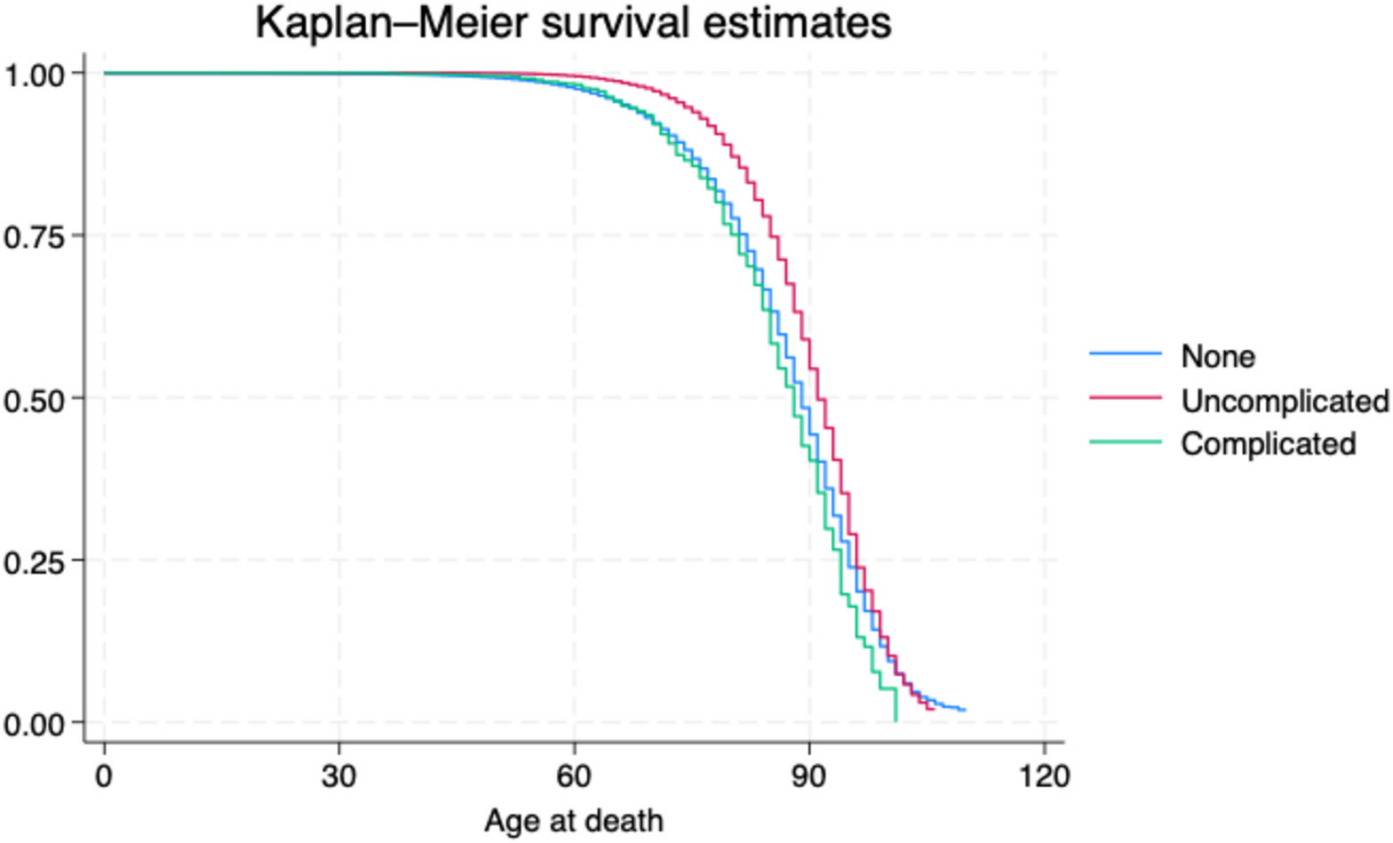
Survival estimates for patients with diverticular disease. Survival is considered in a Kaplan–Meier estimate model the survival probability of patients without colonic diverticular disease (blue), divided by the number of patients at risk, each for uncomplicated (red) and complicated (green) colonic diverticular disease. This is graphed according to age at death in years (0–120).

Controlling for age at first contact and sex yielded a decreased risk of mortality for uncomplicated (HR = 0.61, 95% CI 0.56–0.63; *p* < 0.001), but still two times the associated mortality risk for complicated diverticulitis (HR = 1.17, 95% CI 1.05–1.32; *p* = 0.006). Further controlling for all other potentially confounding variables still yields a similar odds ratio estimate for uncomplicated diverticulitis (HR = 0.65, 95% CI 0.63–0.66; *p* < 0.001) and complicated diverticulitis (HR = 1.18, 95% CI 1.05–1.32; *p* = 0.006) (Figure 2).

## 5 Discussion

We set out to evaluate whether colonic diverticular disease is associated with excess mortality over a prolonged period in a general practice healthcare‐seeking population. Considering potential confounders, we were able to show in this study that overall colonic diverticular disease is not associated with excess mortality over the study period and that age‐related comorbidities likely account for the increased risk.

We further separated the diverticulitis cohort into those with uncomplicated and complicated disease and confirmed that complicated diverticulitis has a higher mortality‐associated excess than uncomplicated diverticulitis despite controlling for age, sex, and other potential confounders.

Patients with diverticulosis and uncomplicated diverticular disease were found to have a lower mortality risk than those without diverticular disease. We can speculate that as these were investigated cases, the lower mortality may reflect previous colonoscopy and removal of incidental polyps or identification of early colonic cancers. We also cannot exclude sociodemographic factors such as those who presented for investigation coming from a higher socioeconomic background and, therefore, being generally healthier. On the other hand, as expected, complicated diverticulitis was associated with a higher mortality regardless of other factors.

In a review of the current literature (Supporting Information S2), four mortality studies in diverticular disease were identified (2009–2022) [3, 6, 19, 20]. Humes et al. in 2009 [20] reported that, in a general practice cohort, patients with perforated diverticular disease were twice as likely to die than the general public, even when corrected for age, sex, and comorbidities. The highest mortality rates occurred in this cohort within the first 3 months following perforation. In the second study published in 2012 [6], Humes et al. reported on mortality within the first year of diagnosis between those with acute and complicated diverticulitis. Overall, when controlled again for age, sex, and comorbidities, mortality in hospital‐reported patients was 1.5 times that of general practice patients when compared with the general population. Excess mortality occurred mainly within the first year post‐diagnosis, especially in those patients with complications of abscess and perforation (15.5%). Interestingly, this study showed that the majority of patients with complicated colonic diverticulitis had no reported prior medical history of acute diverticulitis. The third study by Hunt et al. [19] obtained data from the World Health Organization (WHO) Vital Statistics Registry Mortality Database. Overall, the 58‐nation study indicated that the average age‐adjusted mortality rate from diverticular disease was 0.51 ± 0.31 per 100 000 (range 0.11–1.75/100 000). This study was predominantly concerned with looking at patterns of mortality increase and decrease within developed and developing nations, specifically in proportion to increasing rates of obesity (average % of incidental overweight adults: 0.53 ± 0.10 per year; *p* < 0.0001). Overall, developing nations had an increasing colonic diverticular disease mortality burden (0.027 ± 0.024/100 000 per year) than developed nations (0.005 ± 0.025/100 000 per year; *p* = 0.001). The present data are consistent with the most recent findings of Cameron et al. [3], who assessed the risk of mortality in colonic diverticular disease patients within the Swedish National Healthcare Registers with inflammation observed at histopathology. The study reported that in a Swedish cohort, colonic diverticular disease overall is associated with excess mortality compared with reference individuals in a general population cohort over a minimum 10‐year period, with the highest mortality in the first year of follow‐up after diagnosis. Individuals with colonic inflammation at histopathology and a diagnosis of colonic diverticula had a 36% increased risk of mortality (95% CI = 1.33–1.38), especially in the first‐year post‐diagnosis (HR = 2.18; 95% CI = 2.05–2.32). In this study, the hazard ratio was not controlled for age and sex. Until the present study, diverticular disease has been thought to be associated with overall increased mortality, with the highest rates of mortality occurring within the first year post‐diagnosis.

The current study provides alternative evidence that, overall, patients with colonic diverticular disease, when adjusted for age, sex, and comorbidities, are not at an increased mortality risk compared with reference individuals in a general population cohort. However, when the diverticulitis cohort was subgrouped into uncomplicated and complicated, we observed that patients with complicated diverticulitis were at almost two times increased risk of mortality than their reference counterparts.

In Australia, 0.2% of all deaths were attributed to diverticular disease [3]. In the United States, it has been reported that diverticular disease accounts for 0.9 deaths per 1 000 000 adults [21]. Those who do develop complications from diverticular disease (perforation, abscess, stricture, obstruction, or fistulas) usually require medical intervention [3]. Patients who require admission and surgical management have a higher mortality risk through surgical complications, sepsis, and hemorrhage [3, 22]. A recent Swedish cohort study identified that compared with diverticula‐free patients, diverticulitis sufferers had a four times higher mortality rate within 100 days of their hospital admission (HR 4.44, 95% CI 4.26–4.63) [4].

It is reassuring that uncomplicated diverticular disease is not inherently associated with increased mortality once confounding has been considered as per the present results. Patients who present with acute or uncomplicated diverticulitis are usually managed conservatively, with approximately 80% of these patients reported to be treated successfully [22]. Unfortunately, approximately 20% of patients do not respond to conservative management and progress to complicated disease.

This study had several strengths. By adjusting for age and sex, this study was able to show that patients with colonic diverticular disease are not at increased risk of mortality compared with their nondiverticular counterparts; adjusting for comorbidities did not change the results. The use of a general practice longitudinal database captured the patient's long‐term medical outcomes. To date, data on diverticular disease, even population data, have been limited to hospital records that pertain to patients presenting when symptomatic. As patients are assigned a GP in the United Kingdom and the population is fully covered, a strength of this study design is the ability to capture, in part at least, a representation of patients with colonic diverticula and assess the risk of mortality.

Our study has several limitations. Its primary aim was to evaluate the association between diverticular disease and mortality over a prolonged period using GP cohort data, starting from the first mention of the condition in patient records. The study did not include longitudinal follow‐up or assess surgical interventions over time. Additionally, cause‐of‐death data were unavailable. Inconsistencies in recording symptoms and identifying colonic diverticula across physicians present a potential weakness. The control group (*n* = 1 234 739) was significantly larger than the cohort group (*n* = 38 389), which could introduce selection bias. However, as both groups were derived from the same background population, this risk seems minimal. The Read codes (K57, K57.90) used do not exclusively identify colonic diverticular disease and may include rare cases of small bowel or duodenal diverticulosis. However, given the low prevalence of these conditions, the impact on our findings is likely minimal. Undiagnosed diverticulosis cases in the control group may have inflated the mortality rate, potentially reducing the observed association between diverticular disease and mortality. Nonetheless, we identified higher unadjusted mortality in diverticular disease cases, indicating that any bias was minor. Mortality rates in our controls (9.1% over 10–16 years) and diverticular disease cases (15.9%) were consistent with published UK data. For example, Humes et al. [20] reported mortality rates of 19.4% (controls) and 31.1% (cases) over 10–15 years, whereas Humes and West [6] found similar rates (23% and 35.3%, respectively). Survival probabilities were comparable across studies, with the highest mortality incidence occurring in the first year, aligning with nondiverticular controls thereafter.

Although large control groups can sometimes introduce bias, such as dilution of effect, underestimating confounding, or selection bias, these issues were unlikely in our study because of the shared population background of the groups.

Colonic diverticulosis is often a secondary disease diagnosed during specialist colon examination for other pathology, or as reported from imaging or observation during surgical procedures, so under‐reporting bias is possible. Many of the Read codes/Medcodes did differentiate complications of colonic diverticula, but they did not delineate patients with reoccurring or surgical interventions, nor did they delineate between Hinchey classifications preventing subclassification of complicated cases. Patients who may or may be immunosuppressed were unable to be captured for separate analysis. Another potential limitation that may have skewed our study was the use of a cohort originally recruited for a functional GI disease case/control study, but this is a reasonable sample to study diverticular disease even though it is not a random population sample, as confounding by diagnosis seems unlikely and a large sample was studied. It must be considered that the mean age of patients with colonic diverticula at first contact with their GP (mean age 54) differed from a very large cohort of nondiverticular controls (mean age 38).

In conclusion, our results suggest that overall colonic diverticular disease at baseline is associated with excess mortality, consistent with community sample findings. However, further analysis suggests this is largely because of confounding factors. When age, sex, and comorbidities are controlled for in the analyses, uncomplicated colonic diverticulitis is associated with a lower risk of mortality. However, complicated colonic diverticulitis, even when controlled for other diseases, still carries a two times increased risk of mortality.

## Disclosure

This manuscript represents the views of the authors only. The lead author affirms that this manuscript is an honest, accurate, and transparent account of the study being reported; that no important aspects of the study have been omitted; and that any discrepancies from the study as planned (and, if relevant, registered) have been explained.

## Ethics Statement

This project was provided ethics approval under National Health Service (UK) South‐East Multicentre Research Ethics Committee (Ref: 20/SC/0011) March 20, 2020. Patient data are anonymized, and all data provided under ethics have no identifiable information pertaining to, or linking to, patients.

## Conflicts of Interest

Brown University, Agency for Health Care Research and Quality (fiber and laxation) (2024), UptoDate (section editor) Mayo Clinic Proceeding Associate Editor (2023) Rome Foundation (member gastroduodenal committee) (present) Biocodex (FD diagnostic tool) (present) Microba (consulting microbiome ‐ commencing 2025) Comvita Manuka Honey (FD trial consulting) (2025). Outside the submitted work. In addition, Dr. Talley has a patent Nepean Dyspepsia Index (NDI) 1998, a patent Licensing Questionnaires Talley Bowel Disease Questionnaire licensed to Mayo/Talley, “Diagnostic marker for functional gastrointestinal disorders” Australian Provisional Patent Application 2021901692, ”Methods and compositions for treating age‐related neurodegenerative disease associated with dysbiosis” US Patent Application No. 63/537,725.

## Supporting information


**Supporting Information S1.** Supporting information

**Supporting Information S2.** Supporting information

## References

[1] F. Skoldberg , J. Granlund , A. Discacciati , F. Hjern , P. T. Schmidt , and O. Olen , “Incidence and Lifetime Risk of Hospitalization and Surgery for Diverticular Disease,” British Journal of Surgery 106, no. 7 (2019): 930–939, 10.1002/bjs.11143. 31012495

[2] M. Rabie , H. Fowler , N. N. Dudi‐Venkata , et al., “Diverticulitis Management, a Snapshot Collaborative Audit Study (DAMASCUS): Protocol for an International, Multicentre, Prospective Observational Study,” Colorectal Disease 23, no. 8 (2021): 2182–2188, 10.1111/codi.15699. 33915018

[3] R. Cameron , M. M. Walker , M. Thuresson , et al., “Mortality Risk Increased in Colonic Diverticular Disease: A Nationwide Cohort Study,” Annals of Epidemiology 76 (2022): 39–49, 10.1016/j.annepidem.2022.10.006. 36252891

[4] J. Granlund , F. Sköldberg , F. Hjern , et al., “Mortality in Patients Hospitalized for Diverticulitis in Sweden—A National Population‐Based Cohort Study,” GastroHep 3, no. 3 (2021): 131–140, 10.1002/ygh2.454.

[5] M. von Strauss und Torney , G. Moffa , M. Kaech , et al., “Risk of Emergency Surgery or Death After Initial Nonoperative Management of Complicated Diverticulitis in Scotland and Switzerland,” JAMA Surgery 155, no. 7 (2020): 600–606, 10.1001/jamasurg.2020.0757. 32401298 PMC7221865

[6] D. J. Humes and J. West , “Role of Acute Diverticulitis in the Development of Complicated Colonic Diverticular Disease and 1‐Year Mortality After Diagnosis in the UK: Population‐Based Cohort Study,” Gut 61, no. 1 (2012): 95–100. 21551188 10.1136/gut.2011.238808

[7] H. Al‐Saadi , H. Abdulrasool , and E. Murphy , “Complicated Diverticulitis: Age Distribution, Management and Burden on Health Care,” Cureus 15, no. 2 (2023): e34482, 10.7759/cureus.34709. 36733440 PMC9890077

[8] V. B. Reddy and W. E. Longo , “The Burden of Diverticular Disease on Patients and Healthcare Systems,” Gastroenterología y Hepatología 9, no. 1 (2013): 21–27. PMC3975974 24707230

[9] N. M. Sell , N. P. Perez , C. E. Stafford , et al., “Are There Variations in Mortality From Diverticular Disease by Sex?,” Diseases of the Colon and Rectum 63, no. 9 (2020): 1285–1292, 10.1097/DCR.0000000000001711. 33216498

[10] K. A. Wensaas and A. P. Hungin , “Diverticular Disease in the Primary Care Setting,” Journal of Clinical Gastroenterology 50, no. 1 (2016): S86–S88. 27622376 10.1097/MCG.0000000000000596

[11] M. P. Jones , M. M. Walker , A. C. Ford , and N. J. Talley , “The Overlap of Atopy and Functional Gastrointestinal Disorders Among 23,471 Patients in Primary Care,” Alimentary Pharmacology & Therapeutics 40, no. 4 (2014): 382–391, 10.1111/apt.12846. 24961872

[12] J. D. Lewis , R. Schinnar , W. B. Bilker , X. Wang , and B. L. Strom , “Validation Studies of the Health Improvement Network (THIN) Database for Pharmacoepidemiology Research,” Pharmacoepidemiology and Drug Safety 16, no. 4 (2007): 393–401, 10.1002/pds.1335. 17066486

[13] A. Jaksa , L. Gibbs , S. Kent , et al., “Using Primary Care Data to Assess Comparative Effectiveness and Safety of Apixaban and Rivaroxaban in Patients With Nonvalvular Atrial Fibrillation in the UK: An Observational Cohort Study,” BMJ Open 12, no. 10 (2022): e064662, 10.1136/bmjopen-2022-064662. PMC9577930 36253039

[14] B. T. Blak , M. Thompson , H. Dattani , and A. Bourke , “Generalisability of the Health Improvement Network (THIN) Database: Demographics, Chronic Disease Prevalence and Mortality Rates,” Informatics in Primary Care 19, no. 4 (2011): 251–255, 10.14236/jhi.v19i4.820. 22828580

[15] E. Herrett , S. L. Thomas , W. M. Schoonen , L. Smeeth , and A. J. Hall , “Validation and Validity of Diagnoses in the General Practice Research Database: A Systematic Review,” British Journal of Clinical Pharmacology 69, no. 1 (2010): 4–14, 10.1111/j.1365-2125.2009.03537.x. 20078607 PMC2805870

[16] M. E. Jarbrink‐Sehgal , A. Andreasson , N. J. Talley , L. Agreus , J. Y. Song , and P. T. Schmidt , “Symptomatic Diverticulosis Is Characterized by Loose Stools,” Clinical Gastroenterology and Hepatology 14, no. 12 (2016): 1763–1770, 10.1016/j.cgh.2016.06.014. 27353142

[17] K. Fan , G. D. Eslick , P. M. Nair , et al., “Human Intestinal Spirochetosis, Irritable Bowel Syndrome, and Colonic Polyps: A Systematic Review and Meta‐Analysis,” Journal of Gastroenterology and Hepatology 37, no. 7 (2022): 1222–1234, 10.1111/jgh.15851. 35385602 PMC9545717

[18] K. H. Chuah , W. X. Hian , A. T. Teoh , J. K. Y. Ling , and S. Mahadeva , “Clinical Outcome of Disorders of Gut‐Brain Interaction in Secondary Care: A Longitudinal Study,” Neurogastroenterology and Motility 35, no. 8 (2023): e14602, 10.1111/nmo.14602. 37094070

[19] C. W. Hunt , R. Chaturvedi , L. Brown , et al., “Diverticular Disease Epidemiology: Rising Rates of Diverticular Disease Mortality Across Developing Nations,” Diseases of the Colon and Rectum 64, no. 1 (2021): 81–90, 10.1097/DCR.0000000000001804. 33306534

[20] D. J. Humes , M. Solaymani‐Dodaran , K. M. Fleming , J. Simpson , R. C. Spiller , and J. West , “A Population‐Based Study of Perforated Diverticular Disease Incidence and Associated Mortality,” Gastroenterology 136, no. 4 (2009): 1198–1205, 10.1053/j.gastro.2008.12.054. 19185583

[21] A. F. Peery , “Management of Colonic Diverticulitis,” BMJ 372 (2021): n72, 10.1136/bmj.n72. 33762260

[22] S. Reischl , K. D. Roehl , S. Ziegelmayer , et al., “Radiologic Predictors for Failure of Non‐Operative Management of Complicated Diverticulitis: A Single‐Centre Cohort Study,” Langenbeck's Archives of Surgery 406, no. 7 (2021): 2409–2418, 10.1007/s00423-021-02244-3. PMC8578075 34189654

