# Supplementary material for: Colonic Diverticulosis and Uncomplicated Diverticulitis Are Associated With a Lower Not Higher Risk of Mortality When Confounding Factors Are Held Constant
Source: J Gastroenterol Hepatol. 2025 Mar 17;40(5):1221–9. doi: 10.1111/jgh.16928 (PMC12062918; doi:10.1111/jgh.16928)
Supplement: Supplementary file 1 — Supporting Information S1. Supporting information [file JGH-40-1221-s002.docx]

Supp1

-----------------------------------------------------------------------------------

name: <unnamed>

log: /Users/mq92505007/Dropbox/Newcastle/THIN GI/Variable list.log

log type: text

opened on: 3 Feb 2023, 10:23:27

. desc _all

Variable Storage Display Value

name type format label Variable label

---------------------------------------------------------------------------------------------------------------------------------------------------------------------------------------------------------

person_id long %12.0g

care_site_id int %8.0g

provider_id int %8.0g

gender_code str1 %9s

year_of_birth int %8.0g

first_contact~e str10 %10s

last_contact_~e str10 %10s

first_provide~e byte %8.0g

uid byte %8.0g

year_of_death int %8.0g

month_of_death byte %8.0g

day_of_death byte %8.0g

ethnicity_code str12 %12s

group float %9.0g g

smoker long %8.0g (max) smoker

mfc byte %10.0g

dfc byte %10.0g

date_fc float %9.0g

mlc byte %10.0g

dlc byte %10.0g

date_lc float %9.0g

durncontact float %9.0g Length of MR

age_firstcont~t float %9.0g Age at first contact

age_death float %9.0g Age at death

sex long %8.0g s Sex

vaccination_d~e str10 %10s

vaccine_code str18 %18s

stage byte %8.0g

vacc_date float %9.0g Date of vaccination

vaccnumber float %9.0g v COVID vaccine count

ibs float %9.0g yn IBS

fd float %9.0g yn FD

cons float %9.0g yn Chronic constipation

ibd float %9.0g yn IBD

diverticulosis float %9.0g yn Diverticulosis

diverticulum float %9.0g yn Diverticulum

oesophag float %9.0g yn Oesophagitis

eos_oesophag float %9.0g yn Eosin Oesophagitis

eos_itis float %9.0g yn Eosin gastro/enteritis

chron float %9.0g yn Crohns disease

uc float %9.0g yn Ulcerative colitis

anxiety float %9.0g yn Anxiety

depression float %9.0g yn Depression

hp float %9.0g yn (max) hp

coeliac float %9.0g yn (max) coeliac

gluten_sens float %9.0g yn Gluten sensitivity

asthma float %9.0g yn Asthma

allergy float %9.0g yn Allergy

dmtype1 float %9.0g yn Diabetes type I

dmtype2 float %9.0g yn Diabetes type II

ra float %9.0g yn Rheumatoid arthritis

autoimmune float %9.0g yn Autoimmune disease

lupus float %9.0g yn Lupus erythematosus

scleroderma float %9.0g yn Scleroderma

reflux float %9.0g Reflux

reflux1 float %9.0g (max) reflux1

giinfect float %9.0g Gastroenteritis

duodenitis float %9.0g Duodenitis

gastritis float %9.0g Gastritis

eosinophilia float %9.0g (max) eosinophilia

appendix float %9.0g yn (max) appendix

chole float %9.0g yn (max) chole

hyster float %9.0g yn (max) hyster

fdate_ibs float %9.0g (min) fdate_ibs

fdate_fd float %9.0g (min) fdate_fd

fdate_cons float %9.0g (min) fdate_cons

fdate_ibd float %9.0g (min) fdate_ibd

fdate_diverti~s float %9.0g (min) fdate_diverticulosis

fdate_diverti~m float %9.0g (min) fdate_diverticulum

fdate_eosin float %9.0g (min) fdate_eosin

fdate_oesophag float %9.0g (min) fdate_oesophag

fdate_eos_oes~g float %9.0g (min) fdate_eos_oesophag

fdate_eos_itis float %9.0g (min) fdate_eos_itis

fdate_chron float %9.0g (min) fdate_chron

fdate_uc float %9.0g (min) fdate_uc

fdate_anxiety float %9.0g (min) fdate_anxiety

fdate_depress~n float %9.0g (min) fdate_depression

fdate_hp float %9.0g (min) fdate_hp

fdate_coeliac float %9.0g (min) fdate_coeliac

fdate_gluten_~s float %9.0g (min) fdate_gluten_sens

fdate_asthma float %9.0g (min) fdate_asthma

fdate_allergy float %9.0g (min) fdate_allergy

fdate_dmtype1 float %9.0g (min) fdate_dmtype1

fdate_dmtype2 float %9.0g (min) fdate_dmtype2

fdate_rh float %9.0g (min) fdate_rh

fdate_autoimm~e float %9.0g (min) fdate_autoimmune

fdate_lupus float %9.0g (min) fdate_lupus

fdate_sclerod~a float %9.0g (min) fdate_scleroderma

fdate_reflux float %9.0g (min) fdate_reflux

fdate_reflux1 float %9.0g (min) fdate_reflux1

fdate_giinfect float %9.0g (min) fdate_giinfect

fdate_duodeni~s float %9.0g (min) fdate_duodenitis

fdate_gastritis float %9.0g (min) fdate_gastritis

fdate_eosinop~a float %9.0g (min) fdate_eosinophilia

fdate_appendix float %9.0g (min) fdate_appendix

fdate_chole float %9.0g (min) fdate_chole

fdate_hyster float %9.0g (min) fdate_hyster

ldate_ibs float %9.0g (max) ldate_ibs

ldate_fd float %9.0g (max) ldate_fd

ldate_cons float %9.0g (max) ldate_cons

ldate_ibd float %9.0g (max) ldate_ibd

ldate_diverti~s float %9.0g (max) ldate_diverticulosis

ldate_diverti~m float %9.0g (max) ldate_diverticulum

ldate_eosin float %9.0g (max) ldate_eosin

ldate_oesophag float %9.0g (max) ldate_oesophag

ldate_eos_oes~g float %9.0g (max) ldate_eos_oesophag

ldate_eos_itis float %9.0g (max) ldate_eos_itis

ldate_chron float %9.0g (max) ldate_chron

ldate_uc float %9.0g (max) ldate_uc

ldate_anxiety float %9.0g (max) ldate_anxiety

ldate_depress~n float %9.0g (max) ldate_depression

ldate_hp float %9.0g (max) ldate_hp

ldate_coeliac float %9.0g (max) ldate_coeliac

ldate_gluten_~s float %9.0g (max) ldate_gluten_sens

ldate_asthma float %9.0g (max) ldate_asthma

ldate_allergy float %9.0g (max) ldate_allergy

ldate_dmtype1 float %9.0g (max) ldate_dmtype1

ldate_dmtype2 float %9.0g (max) ldate_dmtype2

ldate_rh float %9.0g (max) ldate_rh

ldate_autoimm~e float %9.0g (max) ldate_autoimmune

ldate_lupus float %9.0g (max) ldate_lupus

ldate_sclerod~a float %9.0g (max) ldate_scleroderma

ldate_reflux float %9.0g (max) ldate_reflux

ldate_reflux1 float %9.0g (max) ldate_reflux1

ldate_giinfect float %9.0g (max) ldate_giinfect

ldate_duodeni~s float %9.0g (max) ldate_duodenitis

ldate_gastritis float %9.0g (max) ldate_gastritis

ldate_eosinop~a float %9.0g (max) ldate_eosinophilia

ldate_appendix float %9.0g (max) ldate_appendix

ldate_chole float %9.0g (max) ldate_chole

ldate_hyster float %9.0g (max) ldate_hyster

fgid float %9.0g yn Any FGID

indx float %9.0g yn

aliment double %9.0g yn Any alimentary Rx

fgidrx double %9.0g yn Any FGID Rx

consrx double %9.0g yn Constipation Rx

diahrx double %9.0g yn Diahhrea Rx

antieos double %9.0g yn Ant-inflammatory Rx

corticoster double %9.0g yn Corticosteroid

antibiot double %9.0g yn Antibiotic

opioid double %9.0g yn Any opioid

ppi double %9.0g yn PPI Rx

propuls double %9.0g yn Propulsive

h2antag double %9.0g yn H2 antagonist

antidep double %9.0g yn Antidepressant

anxiolyt double %9.0g yn Anxiolytic

thromb double %9.0g yn Any antithrombolytic

nsaids double %9.0g yn NSAIDs

statins double %9.0g yn Statins

antihist double %9.0g yn Antihistamine

insulin double %9.0g yn Insulin

other_diab double %9.0g yn Other diab meds

fdate_aliment float %9.0g (min) fdate_aliment

fdate_fgidrx float %9.0g (min) fdate_fgidrx

fdate_consrx float %9.0g (min) fdate_consrx

fdate_diahrx float %9.0g (min) fdate_diahrx

fdate_antieos float %9.0g (min) fdate_antieos

fdate_cortico~r float %9.0g (min) fdate_corticoster

fdate_antibiot float %9.0g (min) fdate_antibiot

fdate_opioid float %9.0g (min) fdate_opioid

fdate_ppi float %9.0g (min) fdate_ppi

fdate_propuls float %9.0g (min) fdate_propuls

fdate_h2antag float %9.0g (min) fdate_h2antag

fdate_antidep float %9.0g (min) fdate_antidep

fdate_anxiolyt float %9.0g (min) fdate_anxiolyt

fdate_thromb float %9.0g (min) fdate_thromb

fdate_nsaids float %9.0g (min) fdate_nsaids

fdate_statins float %9.0g (min) fdate_statins

fdate_antihist float %9.0g (min) fdate_antihist

fdate_insulin float %9.0g (min) fdate_insulin

fdate_other_d~b float %9.0g (min) fdate_other_diab

ldate_aliment float %9.0g (max) ldate_aliment

ldate_fgidrx float %9.0g (max) ldate_fgidrx

ldate_consrx float %9.0g (max) ldate_consrx

ldate_diahrx float %9.0g (max) ldate_diahrx

ldate_antieos float %9.0g (max) ldate_antieos

ldate_cortico~r float %9.0g (max) ldate_corticoster

ldate_antibiot float %9.0g (max) ldate_antibiot

ldate_opioid float %9.0g (max) ldate_opioid

ldate_ppi float %9.0g (max) ldate_ppi

ldate_propuls float %9.0g (max) ldate_propuls

ldate_h2antag float %9.0g (max) ldate_h2antag

ldate_antidep float %9.0g (max) ldate_antidep

ldate_anxiolyt float %9.0g (max) ldate_anxiolyt

ldate_thromb float %9.0g (max) ldate_thromb

ldate_nsaids float %9.0g (max) ldate_nsaids

ldate_statins float %9.0g (max) ldate_statins

ldate_antihist float %9.0g (max) ldate_antihist

ldate_insulin float %9.0g (max) ldate_insulin

ldate_other_d~b float %9.0g (max) ldate_other_diab

inrx float %9.0g

diabetes float %9.0g yn Diabetes (either)

allautoimm float %9.0g yn

agefins float %9.0g

dmtypeI_20 float %9.0g yn Diabetes type I (20-revised)

dmtypeII_20 float %9.0g yn Diabetes type II (20-revised)

dmtypeI_30 float %9.0g yn Diabetes type I (30-revised)

dmtypeII_30 float %9.0g yn Diabetes type II (30-revised)

dmtypeI_40 float %9.0g yn Diabetes type I (40-revised)

dmtypeII_40 float %9.0g yn Diabetes type II (40-revised)

sample float %13.0g sa Sample

. log close

name: <unnamed>

log: /Users/mq92505007/Dropbox/Newcastle/THIN GI/Variable list.log

log type: text

closed on: 3 Feb 2023, 10:23:45

Supp2

**Steps taken to conduct a literature review:**

*28/04/2024*

## **Planning the search (PICO) 1. Research question:**

Current understanding of mortality rates in DD from either a population-based or community-based study

Population of problem: diverticulosis, diverticulitis, diverticular disease

Intervention or exposure: place of residence (community or population source)

Comparison: disease to non-diverticula deaths

Outcome: death, mortality rates

**2. Subject areas** to identify keywords, phrases, synonyms, and alternative spellings:

Topic 1: Diverticular Disease

Keywords: {‘diverticul*’}
 Can be narrowed with: {‘colon*’ OR ‘sigmoid’}

Topic 2: Community or population studies

Keywords: {‘community”; “population”, exclude “hospital’}

Also try health-care setting, general practice

Topic 3: mortality

Keywords: {‘mortality’}

As a full search:

Addition exclusion/inclusions: English language. Humans. Years 2000 to 2024.

## **3. Searches**

i. Did a library search to see if any other reviews on the subject had been undertaken:

a. Search terms :

i. Results for t:(diverticul*) (review) (colon*) (mortality) y:[2000-2024]

Results: **371**

I then further refined the search:
 Review Articles AND English AND Subjects: (diverticulitis, mortality, gastroenterology, review, female, male, diverticular disease, risk factors)

Results: **142**

Of the 142:
 7 discussed pathogenesis in general
 0 discussed mortality specifically
 135 were other subject specific (not related to question) or duplicates
 **0 were on topic**

**Suggest limiting search to titles only**

**Suggested included words:**

**Suggested excluded words:** surgery (surg*), procedure, hospital, post/operat*

ii. Searched systematic review databases to identify similar systematic reviews using search terms as above were relevant and expanded the search to ‘apply related words’.

Cochrane Database of Systematic Reviews (CDSR) PICO search^[[1]](#footnote-1)^: Results: 0

Cochrane Database of Systematic Reviews (CDSR) MeSH search^[[2]](#footnote-2)^: Results: 0

Campbell Library^[[3]](#footnote-3)^: Results: 0

PubMed Clinical Queries^[[4]](#footnote-4)^: (search: (diverticul*) (mortality) (review) (population): category: etiology/broad). Results: 67

Prospero: Results: 0
(search: diverticular AND mortality AND population). Results: 11

**Registration of review in Prospero:**

PROSPERO does not accept scoping reviews, literature reviews or mapping reviews for registration.

iii. I used the search results from i. and ii. to review the titles of publications found, aiming to increase the keywords for searches of databases: any further addition of words or phrases were added to the PICO chart and search parameters (see above).

iv. Literature scoping

Searched: *01/05/2024* as per PRISMA 2009 Flow Diagram (link to below table)

| Search carried out in: SCORPUS <https://www-scopus-com.ezproxy.newcastle.edu.au/> | | | |
| --- | --- | --- | --- |
| **#** | **Searches** | **Results** | **Relevant** |
| 1 | divertic* | 51 437 |  |
| 2 | colon* | 1 295 791 |  |
| 3 | #1 or #2 | 17 582 |  |
| 4 | Mortality (title only) | 200 848 |  |
| 5 | #3 AND #4 | 95 | 14 |

| Search carried out in: MEDLINE via UoN OVID | | | |
| --- | --- | --- | --- |
| **#** | **Searches** | **Results** | **Relevant** |
| 1 | divertic* (Human) | 15 287 |  |
| 2 | colon* | 9 988 |  |
| 3 | #1 AND #2 | 10 446 |  |
| 4 | Mortality | 17 394 |  |
| 5 | #3 AND #4 (Humans) | 67 | 13 |

| Search carried out in: Cochrane: <https://www-cochranelibrary-com.ezproxy.newcastle.edu.au/> | | | |
| --- | --- | --- | --- |
| **#** | **Searches** | **Results** | **Relevant** |
| 1 | divertic* | 83 |  |
| 2 | colon* | 1 065 |  |
| 3 | #1 AND #2 | 36 |  |
| 4 | #3 AND mortality | 11 | 0 |

| Search carried out in: Web of Science: <http://apps.webofknowledge.com.ezproxy.newcastle.edu.au/> | | | |
| --- | --- | --- | --- |
| **#** | **Searches** | **Results** | **Relevant** |
| 1 | divertic* | 27 030 |  |
| 2 | colon* | 993 434 |  |
| 3 | #1 AND #2 | 7 794 |  |
| 4 | #3 AND mortality (Title) | 81 | 19 |

| Search carried out in: CINAHL <https://web-p-ebscohost-com.ezproxy.newcastle.edu.au/ehost/search/advanced?vid=0&sid=22348a90-633d-47fa-b13c-d0e51ca6fc22%40redis> | | | |
| --- | --- | --- | --- |
| **#** | **Searches** | **Results** | **Relevant** |
| 1 | divertic* | 5 307 |  |
| 2 | colon* | 76 505 |  |
| 3 | #1 AND #2 | 1 364 |  |
| 4 | Mortality | 360 910 |  |
| 5 | #3 AND mortality (full text, English) | 20 | 1 |

| Search carried out in: Google Scholar: https://scholar.google.com/ | | | |
| --- | --- | --- | --- |
| **#** | **Searches** | **Results** | **Relevant** |
| 1 | divertic* | 23 300 |  |
| 2 | colon* | 3 100 000 |  |
| 3 | #1 AND #2 | 20 900 |  |
| 4 | #3 AND mortality allintitle | 0 |  |
| 5 | Diverticular AND mortality allintitle | 14 | 4 |

## **4. Screening and eligibility**

*May 2024*

Combination of all references (including grey literature) to one file: = **51**

Duplicates removed: = **20**

Abstract and title screening: **31**

Full text screening against the inclusion and exclusion criteria: **12**

- References checked for updates and full text search done.
- Individual references screened for gaps and subjected to inclusion/exclusion criteria.

| **Inclusion** | **Exclusion** |
| --- | --- |
| English language (or able to be translated) | Non-English (or unable to be translated) |
| Human studies | Animal studies |
| Over age 18/adults | Under 18/children |
| Country or international population, community or general health care population | Post/Surgical or hospital dataset only |

***Inclusion and Exclusion criteria:***

= **4** for extraction

**5. Papers for Data extraction**

*May 2024*

Data extracted into a spreadsheet, with a row for each paper; and extracted data.

Any full texts that upon further reading no longer fit the criteria were further excluded.

*Papers for extraction:*

1. Humes DJ, Solaymani–Dodaran M, Fleming KM, Simpson J, Spiller RC, West J. A population-based study of perforated diverticular disease incidence and associated mortality. Gastroenterology. **2009**;136(4):1198-205.

2. Humes DJ, West J. Role of acute diverticulitis in the development of complicated colonic diverticular disease and 1-year mortality after diagnosis in the UK: population-based cohort study. Gut. **2012**;61(1):95-100.

3. Hunt CW, Chaturvedi R, Brown L, Stafford C, Cauley CE, Goldstone RN, et al. Diverticular Disease Epidemiology: Rising Rates of Diverticular Disease Mortality Across Developing Nations. Dis Colon Rectum. **2021**;64(1):81-90.

4. Cameron R, Walker MM, Thuresson M, Roelstraete B, Skoldberg F, Olen O, et al. Mortality risk increased in colonic diverticular disease: a nationwide cohort study. Ann Epidemiol. **2022**;76:39-49.

*Extracted data from final screening:*

|  | *Timespan* | *Population* | *Data* | *Country/ies* | *DD specifics* | *N=* | *Controls* | *Overall mortality* | *Age/Sex adj.* | *Co-morb. adj.* |
| --- | --- | --- | --- | --- | --- | --- | --- | --- | --- | --- |
| *1* | 1990-2005 | General practice | ICD-10 | UK | Perforated | 953 | Yes | The first 3 months following perforation had the highest mortality rates with a 3-month survival of 86.3%. The first year demonstrated that the disease cohort was nearly 6 times more likely to die than the general population cohort (HR, 5.63 [95% CI: 4.68 – 6.77]). | Done with comorbidities.  Males 382 / females 571  Inc. / 100 000 person years  3.14 (2.89–3.41) / 2.16 (1.95–2.39) | Twice as likely as the general population cohort to die when corrected for age, sex, smoking, and obesity (hazard ratio [HR], 2.21 [95% CI: 1.95–2.50]). However, when stratifying by comorbidity, the adjusted relative risk of death in the first year was highest in those with lowest comorbidity (Charlson group 0: HR, 11.11; 95% CI: 8.06–15.31), but the absolute mortality rates were greatest in those with the highest comorbidity (Charlson group ⬎2: mortality rate, 263.10 per 1000 person-years). These findings were independent of age and sex. |
| *2* | 1990-2007 | General practice (but also hospitalisations – ? excluded) | ICD-10 | UK | Acute -> complic. | 2950 | Yes | Most of the excess mortality occurred in the first year after diagnosis. The 1-year mortality of all cases was 12.8% (377/2932), with the greatest 1-year mortality associated with abscess and perforation at 15.5% (239/1546).  Interesting extra:  This study has shown that most patients with complicated colonic diverticular disease had no prior history of acute diverticulitis, but those with a fistula were most likely to have had at least one prior episode of prior acute diverticulitis. | The disease cohort was more likely to be women than men (63.5% vs 36.5%, χ2 p<0.001). | Overall mortality in HES was 1.5 times that of the general population, controlling for age, sex and comorbidity (HR 1.46, 95% CI 1.25 to 1.71), compared with 1.5 times in cases identified from GPRD (HR 1.54, 95% CI 1.42 to 1.66). |
| *3* | 1994-2016 | WHO | ICD-10 | International | K57: DD of intestine only and obesity | 58 nations 24,796,270 ± 48,733,733 | No | The average age-adjusted mortality rate for diverticular disease was 0.51 ± 0.31/100,000 with a range of 0.11 to 1.75/100,000. During the study period, we noted that 57% of nations had increasing diverticular disease mortality rates, whereas only 7% had decreasing rates. More developed nations (40%) than developing nations (24%) were categorized as having high diverticular disease mortality burden over the time period of the study, and developed nations had higher percentages of overweight adults (58.9 ± 3.1%) than developing nations (50.6 ± 6.7%; p < 0.0001). However, developing nations revealed more rapid increases in diverticular disease mortality (0.027 ± 0.024/100,000 per year) than developed nations (0.005 ± 0.025/100,000 per year; p = 0.001), as well as faster expanding proportions of overweight adults (0.76 ± 0.12% per year) than in already developed nations (0.53 ± 0.10% per year; p <0.0001). | The average age-adjusted mortality rate from diverticular disease was 0.51 ± 0.31 per 100,000 (range 0.11–1.75/100,000). |  |
| *4* | 1987-2017 | Swedish TPR & COD Register | ICD-8, 9, 10 | Sweden | DD & inflamm. | 97 8850 | Yes, and siblings | The cumulative mortality at 30 days after diverticular disease diagnosis was 1.6% (n = 1158), at 90 days 2.8% (n = 2776), and at 365 days 5.9% (n = 5733).  - overall that the risk of death was increased by 27%. In absolute terms, there was one extra death in 100 diverticular disease patients followed for 1 year compared to reference individuals (on average 4.4 vs. 3.4 deaths). During follow-up, there were 32,959 deaths in individuals with colonic diverticular disease (44/1000 person-years) compared with 127,153 in matched reference individuals (34/1000 person-years), resulting in an HR of 1.27 (95%CI 1.25–1.29) | The male to female ratio was matched between cases and reference individuals, (41/59%, 41/59% respectively), and the sibling ratio was 50/50. | Only 789 (2.4%) of all deaths had diverticulitis as the underlying cause of death.  Individually, death from colorectal cancer contributed to the crude HR by having the highest HR 8.84 (7.50–10.43), although after the first year of follow-up this decreased and at the 5-year follow-up timepoint, results were no longer statistically significant.  Individuals with diverticular disease whose colorectal histology had shown inflammation were at a + 36% increased risk of death (95%CI = 1.33–1.38) (eTable 3), with the highest HR seen in the first year of follow-up (HR = 2.18; 95%CI = 2.05–2.32). HRs for death were lower in diverticular disease patients with normal colorectal mucosa (HR = 1.21; 95%CI = 1.18–1.24) (P < .001 for interaction) but remained statistically significant also beyond 5 years after diverticular diagnosis |

**The extracted papers were used within the body and discussion of the manuscript titled:** Colonic diverticular disease is associated with a lower not higher risk of mortality when confounding factors are held constant.

**Authors:**

Raquel Cameron^1-3^, Michael Jones^2,4^, Guy D. Eslick^2-3^, Nicholas J. Talley, MD PhD^1-3^

College of Health, Medicine and Wellbeing, University of Newcastle, Newcastle, Australia
2. NHMRC Centre for Research Excellence in Digestive Health
3. Hunter Medical Research Institute, Newcastle, Australia
4. Macquarie University, School of Psychological Sciences, North Ryde, Australia

1. <https://www.cochranelibrary.com/en/advanced-search/pico> [↑](#footnote-ref-1)
2. <https://www.cochranelibrary.com/advanced-search/mesh> [↑](#footnote-ref-2)
3. <https://www.campbellcollaboration.org/> [↑](#footnote-ref-3)
4. <https://www.ncbi.nlm.nih.gov/pubmed/clinical> [↑](#footnote-ref-4)
